# Associations of maternal diet and nutritional status with offspring hepatic steatosis in the Avon longitudinal study of parents and children

**DOI:** 10.1186/s40795-021-00433-3

**Published:** 2021-07-08

**Authors:** Ahlia Sekkarie, Jean A. Welsh, Kate Northstone, Aryeh D. Stein, Usha Ramakrishnan, Miriam B. Vos

**Affiliations:** 1 Nutrition and Health Sciences Program, Laney Graduate School, Emory University, 1518 Clifton Rd NE, Atlanta, GA 30322 USA; 2 Department of Pediatrics, Emory School of Medicine, Atlanta, GA 30322 USA; 3 Population Health Science, Bristol Medical School, Bristol, BS8 2BN UK; 4 Hubert Department of Global Health, Rollins School of Public Health, Emory University, Atlanta, GA 30322 USA

**Keywords:** NAFLD, Obesity, Pregnancy, Diabetes, Longitudinal, ALSPAC

## Abstract

**Background:**

Priming for cardiometabolic diseases, including non-alcoholic fatty liver disease (NAFLD), is hypothesized to begin in utero. The primary objective of this study is to determine whether there is an association between maternal nutritional status and offspring NAFLD.

**Methods:**

Data come from the Avon Longitudinal Study of Parents and Children (ALSPAC) in the UK. The analytic sample included 3353 participants who had maternal information on pre-pregnancy BMI, gestational weight gain, diabetes, and free sugar intake as percent of total energy and were assessed for mild-severe hepatic steatosis at 24 years by transient elastography (controlled attenuation parameter score ≥ 248 dB/m). Multiple logistic regression was used to evaluate the association between maternal factors and offspring hepatic steatosis at 24 years.

**Results:**

In confounder-adjusted models the independent associations for each maternal factor with mild to severe vs low hepatic steatosis at 24 years were: pre-pregnancy overweight (OR: 1.84, 95%CL: 1.43–2.38) or obesity (OR: 2.73, 95%CL: 1.84–4.03), more than recommended gestational weight gain (OR: 1.30, 95%CL: 1.04–1.64), diabetes (OR: 1.39, 95%CI: 0.87, 2.21), and high free sugar intake during pregnancy (OR: 1.04, 95% CI: 0.82, 1.33). These associations were largely mediated by BMI at 24 years, but not by birthweight or breastfeeding.

**Conclusions:**

Our results suggest that maternal nutritional status is associated with the development of NAFLD in their adult offspring, although the relationship is largely mediated by offspring BMI in adulthood.

**Supplementary Information:**

The online version contains supplementary material available at 10.1186/s40795-021-00433-3.

## Background

Non-alcoholic fatty liver disease (NAFLD) is defined as having excessive hepatic steatosis in the absence of other liver diseases, extreme alcohol intake, or medication-induced steatosis [1]. Established risk factors for NAFLD include increasing age, male sex, high sugar diets, and obesity. In children, the prevalence of NAFLD has increased considerably over recent decades in parallel with the rise of obesity [2]. This is concerning because pediatric NAFLD can progress to nonalcoholic steatohepatitis (NASH), which is characterized by inflammation, as well as cirrhosis and end stage liver disease in adulthood [3]. NAFLD is also associated with increased risk of diabetes and cardiovascular disease [4, 5].

The clinical manifestation of NAFLD in children and the presence of steatosis at birth in some newborns suggest that its origins may lie in utero [6–9]. The Developmental Origins of Health And Disease (DOHAD) paradigm posits that environmental factors during fetal and early life program the risk of metabolic diseases including NAFLD [10, 11]. Currently, the fetal environment primarily occurs in a context of high prevalence of maternal obesity as well as high sugar intake that continues to increase globally [12, 13]. Studies in animal models have shown that maternal diets high in sugar and fat predispose offspring to developing NAFLD phenotypes [14–19]. In humans, a maternal diet high in added sugar intake during pregnancy has been associated with an increased risk of obesity in children [20, 21]. While no human studies have directly looked at the association between maternal energy rich diets and offspring NAFLD explicitly, pre-pregnancy obesity and overweight, maternal diabetes, and gestational weight gain have all been associated with increased hepatic fat in infants and adolescents [7, 8, 22–24]. There is also evidence that breastfeeding has a protective effect on hepatic steatosis in individuals that were exposed to excess nutrition in utero [22]. However, whether outcomes are dependent on child sex and the impact of childhood factors such as adiposity in mediating the outcome have been inconsistent. It is also not known whether this association extends into adulthood.

The overall aim of this paper is to explore whether the associations between maternal nutritional status and offspring NAFLD extend into adulthood in the Avon Longitudinal Study of Parents and Children (ALSPAC), based in the UK. Secondary aims are to determine whether there is sexual dimorphism in these associations and whether the associations are mediated by birthweight, breastfeeding, or BMI at time of outcome.

## Methods

### Study design & population

We used data from the Avon Longitudinal Study of Parents and Children, a population-based birth cohort study that has previously been described in detail [25–27]. In summary, ALSPAC enrolled 14,541 pregnant women in the greater Bristol, UK area with expected delivery dates between 1st April 1991 and 31st December 1992. At the age of seven, attempts were made to boost the sample, resulting in a total of 15,454 pregnancies and 14,901 children alive at the age of one year. Clinical, dietary, and demographic information was collected from the mothers starting in pregnancy. When the offspring were 24 years of age, 10,018 participants were invited to a clinic visit known as Focus@24, which included the collection of biological samples and anthropometric measures. Data from the 24-year clinic were collected and managed using REDCap electronic data capture tools hosted at the University of Bristol [28, 29]. The study website contains details of all the data that are available through a fully searchable data dictionary and variable search tool [30].

Ethical approval for the study was obtained from the ALSPAC Ethics and Law Committee and the Local Research Ethics Committees. Informed consent for the use of collected data via questionnaires and clinics was obtained from participants following the recommendations of the ALSPAC Ethics and Law Committee at the time.

### Assessment of maternal nutrition

#### Pre-pregnancy BMI

Pre-pregnancy weight and height were self-reported via postal questionnaire and were used to calculate body mass index (BMI) as weight in kilograms divided by height in meters squared [31]. We classified BMI according to World Health Organization categories as underweight (< 18.5 kg/m^2^), normal (18.5 to < 25 kg/m^2^), overweight (25 to < 30 kg/m^2^), and obese (≥ 30 kg/m^2^) [32].

#### Gestational weight gain (GWG)

Gestational weight and the corresponding gestational age and date were abstracted by trained research midwives from obstetric medical records [33, 34]. As previously described, weight gain was predicted from linear spline models as the difference between predicted weight at time of delivery and weight at gestational age of 0 weeks [33, 34]. Women were then categorized into three categories according to Institute of Medicine (IoM) recommendations: adequate, less than, and more than recommended GWG. Recommended weight gain is 12.5–18 kg for underweight; 11.5–16 kg for normal weight; 7–11.5 kg for overweight; and 5–9 kg for obese women [35].

#### Maternal diabetes

Due to limited sample size, maternal diabetes was defined as a composite variable that included pre-existing diabetes assessed by self-reported questionnaire at time of recruitment, gestational diabetes mellitus (GDM), and glycosuria (≥ 13.9 mmol/l on at least two occasions during pregnancy) abstracted from medical records, as previously described [24].

#### Maternal free sugar intake

Maternal intake of 43 food groups was assessed by food frequency questionnaire at 32 weeks gestation. The questionnaire can be found on the study website [36]. This information was combined with nutrient information on standardized portion sizes to calculate macronutrient intakes, as described in detail previously [37]. Non-milk extrinsic sugars (NMES) were calculated by deducting sugars from milk and fruits and vegetables (contained within cellular walls) from total sugars [38]. This is equivalent to the definition of free sugars which includes isolated sugars added during food preparation and manufacturing (added sugars) as well as sugars present in unsweetened fruit juices, fruit concentrates, or honey and other syrups [39]. We calculated the percent of total energy consumed as free sugars by dividing each participant’s estimated non-milk extrinsic sugar intake by total energy intake and categorized it into tertiles (hereafter referred to as percent free sugar).

### Assessment of liver outcomes

At 24 years old, participants were assessed by transient elastography for non-invasive quantification of liver steatosis and fibrosis (FibroScan® 502 Touch, Echosens, Paris, France). Individuals with an active medical implant (e.g. pacemaker), liver ascites, or who were pregnant were excluded from the liver scan. Participants were asked to fast overnight or for at least six hours prior to transient elastography [40]. Transient elastography provides a controlled attenuation parameter (CAP) measure of steatosis and a measure of liver stiffness to quantify fibrosis. Manufacturer and machine indications were used to conduct the scan. Ten readings were required for each patient to derive a CAP score and fibrosis result. CAP values outside the 100–400 dB/m range were considered invalid and coded as missing. Median fibrosis results greater than or equal to 15 kPa or with an interquartile range (IQR) to median ratio greater than or equal to 30% were considered invalid and coded as missing.

We categorized participants into two categories of steatosis based on CAP score cut-off values derived from a meta-analysis by Karlas, et al.: low (< 248 dB/m, < 11% steatosis) vs mild to severe (248–400 dB/m, ≥11% steatosis) [41]. In sensitivity analysis we also categorized steatosis as low to moderate steatosis (< 279 dB/m, < 66% steatosis) vs severe steatosis (279–400 dB/m, ≥66% steatosis). We categorized fibrosis into two groups. The first group included those with no fibrosis or portal fibrosis without septa (F0-F1, < 7.9 kPA) and the second group included those with any fibrosis: portal fibrosis, septa, or cirrhosis (F2-F4, > 7.9 kPA) [42].

### Covariates

Maternal age at delivery was derived from the mother’s report of her date of birth and the infant’s date of birth. Offspring birthweight and sex were extracted from medical records. We used highest level of maternal education reported during pregnancy as a proxy for socioeconomic status [43]. Mothers self-reported one of five categories: None/CSE (certificate of secondary education), Vocational (vocational courses after 16 years of age), O (ordinary level exams at 16 years), A (optional advanced level exams at 18 years), and University degree and above [44]. Mothers self-reported smoking (1st and 3rd trimester) and alcohol intake (1st, 2nd, and 3rd trimester); we categorized each as none vs any at any time point in pregnancy. Physical activity was assessed via questionnaire in the first trimester and was categorized as at least once per week versus less than weekly. Infant birthweight was abstracted from medical records and we classified it as low (< 2500 g), normal, and high (> 3999 g) [45]. Breastfeeding duration was assessed from maternal reports when the child was 15 months old and categorized as never, less than 3 months, 3 to 6 months, and greater than 6 months. Offspring BMI at 24 years was calculated from height and weight collected by standardized clinic protocols and categorized as described above for the mothers. We defined hazardous alcohol consumption as an Alcohol Use Disorder Identification Test for Consumption (AUDIT-C) score greater than or equal to four in women and five in men [46, 47].

### Inclusion/exclusion

We included all participants who attended the Focus@24 visit and had valid transient elastography measures. We excluded participants from twin pregnancies and those who were missing all four maternal exposures (pre-pregnancy BMI, gestational weight gain, diabetes, and percent free sugars). In models with percent free sugar as the primary exposure, we additionally excluded those with mothers who had pre-existing or gestational diabetes since this could alter their consumption patterns.

### Statistical analysis

We conducted statistical analyses in SAS version 9.4 (Cary, NC). We calculated median values and IQR for continuous variables and counts and percentages for categorical variables, for the full sample and stratified by level of hepatic steatosis (low vs mild to severe) at 24 years. Wilcoxon rank sum (equal variance) and Kolmogrov-Smirnov (unequal variance) tests were used to compare differences in continuous variables across hepatic steatosis levels. Chi-squared tests were used to compare differences between categorical variables. All results were interpreted based on the size, direction, and CIs of the effect estimates and not focused on statistical significance [48].

**Fig. 1:**
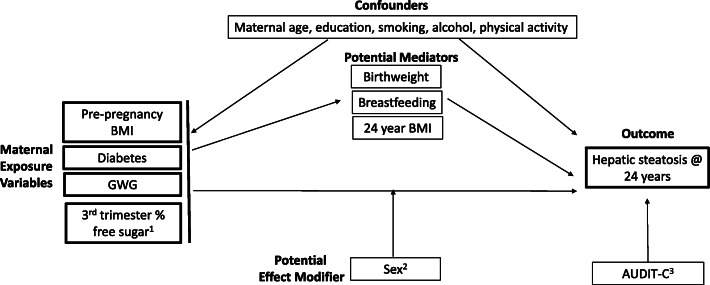
Conceptual model relating maternal exposures to mild to severe hepatic steatosis at 24 years in the ALSPAC cohort. ^1^In models with % free sugars as the primary exposure, those with pre-existing or gestational diabetes were excluded. Total energy intake was also added as a confounder in these models. ^2^Sex was considered a confounder if the interaction term was not significant at *p* < 0.05. ^3^Hazardous alcohol intake, quantified by AUDIT-C score, was adjusted for in sensitivity analysis. Abbreviations: BMI = body mass index, GWG = gestational weight gain, AUDIT-C = alcohol use disorders identification test consumption

Figure 1 shows a simple conceptual model on which our modeling strategy was based. We assessed each exposure for potential effect modification by sex by including an interaction term between sex and the exposure. If this term was not significant for all exposures, sex was added as a term in the adjusted models. The primary outcome was mild to severe hepatic steatosis. Unadjusted binary logistic regression was conducted for each exposure in the model 1 series: pre-pregnancy BMI, maternal diabetes, gestational weight gain, and percent free sugar intake.

In the model 2 series we adjusted for confounders: maternal age, highest level of maternal education, maternal smoking in pregnancy, alcohol intake in pregnancy, and physical activity in pregnancy. Model 2a included pre-pregnancy BMI, maternal diabetes, and gestational weight gain. Model 2b, which focused on percent free sugar exposure, additionally adjusted for total energy intake.

If the association between exposure and steatosis remained after confounder adjustment, we considered the following potential mediators: a) birthweight, b) breastfeeding, and c) offspring BMI at 24 years.

A sensitivity analysis was performed with the primary outcome defined as severe steatosis. In addition, to understand the impact of alcohol consumption, an important predictor of hepatic steatosis, we added hazardous alcohol intake at 24 years as a covariate in the models.

## Results

**Fig. 2:**
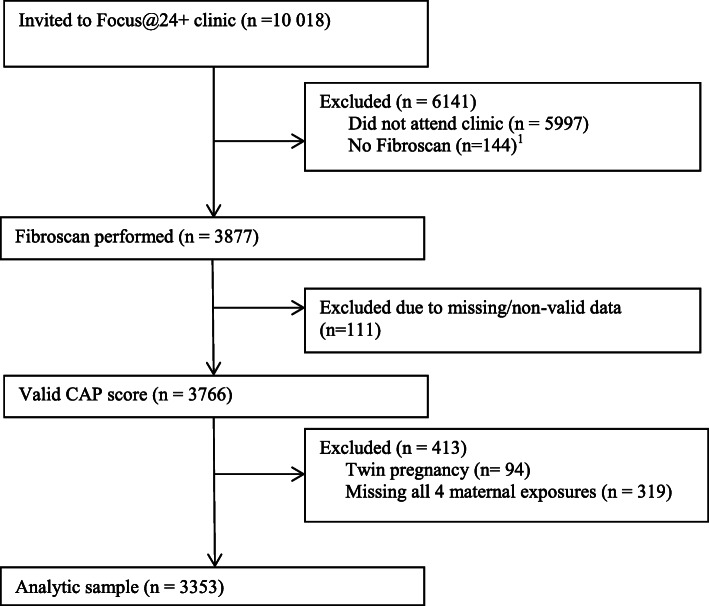
Data flow chart for ALSPAC cohort analysis. Of the 10,018 ALSPAC participants invited to attend the Focus@24+ visit, 3877 had Fibroscan for hepatic steatosis measure performed. Of these, 3766 had a valid CAP score. After exclusions for twin pregnancies and those missing all four maternal exposures, the final sample size was 3353. ^1^No consent for liver scan, not eligible, or excluded due to active implant, liver ascites, or pregnancy. Abbreviations: CAP = controlled attenuation parameter

Of the 10,018 ALSPAC participants who were invited to attend the Focus@24+ visit, 3877 (39%) participants had FibroScan® performed. Of these, 3766 (97%) participants had a valid CAP score. After exclusions for twin pregnancies and those missing all four maternal exposures, our sample size was 3353 (86% of those with FibroScan®, Fig. 2). Demographic characteristics of the sample overall and stratified by offspring hepatic steatosis level are presented in Table 1. Approximately 20% of the offspring had hepatic steatosis at 24 years. Among the mothers, 16.7% were overweight or obese pre-pregnancy, less than 4% had diagnosed pre-existing diabetes, gestational diabetes or glycosuria, and over half had greater than recommended gestational weight gain. Those in the lowest tertile of percent free sugar intake had values ranging from 1.3 to 10.4%, middle to 14.3%, and upper tertile to 42.2%. The median maternal age was 29 years (IQR: 26.0, 32.0). Lower maternal education, smoking during pregnancy, and no breastfeeding were more prevalent in the mothers of offspring with steatosis. Most participants were female (62.2%), with male sex more strongly associated with hepatic steatosis. Over a third (37.5%) of participants were overweight or obese at age 24. Only 2.4% had hepatic fibrosis.

**Table 1:** Demographic and clinical factors by hepatic steatosis^a^ at 24 years in the ALSPAC cohort (*n* = 3353)

Median (IQR) or n (%)	Total (n = 3353)	Low (***n*** = 2662;79.4%)	Mild-Severe (***n*** = 691; 20.6%)	P-Value^d^
**Maternal & Infant Factors**				
**Maternal age**	29.0 (26.0, 32.0)	29.0 (27.0, 32.0)	29.0 (26.0, 32.0)	**< 0.001**
**Maternal Highest Education**				**< 0.001**
Missing	65 (1.9%)	60 (2.3%)	5 (0.7%)	
CSE/None	313 (9.3%)	236 (8.9%)	77 (11.1%)	
Vocational	246 (7.3%)	178 (6.7%)	68 (9.8%)	
O-level	1113 (33.2%)	861 (32.3%)	252 (36.5%)	
A-level	939 (28.0%)	766 (28.8%)	173 (25.0%)	
Degree	677 (20.2%)	561 (21.1%)	116 (16.8%)	
**Pregnancy physical activity**				0.828
Missing	253 (7.5%)	201 (7.6%)	52 (7.5%)	
No	2098 (62.6%)	1672 (62.8%)	426 (61.6%)	
Yes	1002 (29.9%)	789 (29.6%)	213 (30.8%)	
**Pregnancy smoking**				**0.027**
Missing	11 (0.3%)	9 (0.3%)	< 5^c^	
No	2799 (83.5%)	2241 (84.2%)	558 (80.8%)	
Yes	543 (16.2%)	412 (15.5%)	131 (19.0%)	
**Pregnancy alcohol intake**				
Missing	10 (0.3%)	9 (0.3%)	< 5^c^	**0.002**
No	1823 (54.4%)	1410 (53.0%)	413 (59.8%)	
Yes	1520 (45.3%)	1243 (46.7%)	277 (40.1%)	
**Maternal pre-pregnancy BMI**	22.0 (20.5, 24.1)	21.8 (20.4, 23.7)	22.7 (20.9, 25.7)	**< 0.001**
Missing	255 (7.6%)	208 (7.8%)	47 (6.8%)	
Underweight	127 (3.8%)	109 (4.1%)	18 (2.6%)	
Normal	2411 (71.9%)	1968 (73.9%)	443 (64.1%)	
Overweight	420 (12.5%)	294 (11.0%)	126 (18.2%)	
Obese	140 (4.2%)	83 (3.1%)	57 (8.2%)	
**Maternal diabetes**				0.314
Missing	86 (2.6%)	70 (2.6%)	16 (2.3%)	
None	3140 (93.6%)	2500 (93.9%)	640 (92.6%)	
Pre-existing	11 (0.3%)	7 (0.3%)	< 5^c^	
Gestational	13 (0.4%)	10 (0.4%)	< 5^c^	
Glycosuria	103 (3.1%)	75 (2.8%)	28 (4.1%)	
**Gestational weight gain**				**0.002**
Missing	321 (9.6%)	262 (9.8%)	59 (8.5%)	
Recommended	900 (26.8%)	746 (28.0%)	154 (22.3%)	
< Recommended	370 (11.0%)	299 (11.2%)	71 (10.3%)	
> Recommended	1762 (52.5%)	1355 (50.9%)	407 (58.9%)	
**Total energy intake (kJ/day)**^b^	7127 (6000, 8361)	7162 (6050, 8371)	6977 (5813, 8237)	**0.009**
**Free sugars (g/day)**^b^	51.7 (36.8, 70.8)	52.3 (36.9, 71.3)	50.1 (36.7, 69.5)	0.189
**% Free sugars**^b^	0.12 (0.10, 0.16)	0.12 (0.10, 0.16)	0.12 (0.09, 0.16)	0.871
**Sex (% male)**	1276 (38.1%)	946 (35.5%)	330 (47.8%)	**< 0.001**
**Birthweight (g)**	3440 (3130, 3760)	3423 (3120, 3750)	3483 (3180, 3805)	**0.014**
Missing	43 (1.3%)	32 (1.2%)	11 (1.6%)	0.409
Low	106 (3.2%)	84 (3.2%)	22 (3.2%)	
Normal	2783 (83.0%)	2223 (83.5%)	560 (81.0%)	
High	421 (12.56%)	323 (12.1%)	98 (14.2%)	
**Breastfeeding**				**0.011**
Missing	230 (6.9%)	185 (6.9%)	45 (6.5%)	
Never	509 (15.2%)	381 (14.3%)	128 (18.5%)	
< 3 months	640 (19.1%)	509 (19.1%)	131 (19.0%)	
3–5 months	543 (16.2%)	418 (15.7%)	125 (18.1%)	
≥ 6 months	1431 (42.7%)	1169 (43.9%)	262 (37.9%)	
**Adult characteristics at 24 years**				
**BMI (kg/m**^**2**^**)**	23.8 (21.5, 26.9)	23.0 (21.0, 25.4)	28.6 (25.2, 33.1)	**< 0.001**
Missing	31 (0.9%)	23 (0.9%)	8 (1.2%)	**< 0.001**
Underweight	103 (3.1%)	99 (3.7%)	< 5^c^	
Normal	1961 (58.5%)	1807 (67.9%)	154 (22.3%)	
Overweight	842 (25.1%)	592 (22.2%)	250 (36.2%)	
Obese	416 (12.4%)	141 (5.3%)	275 (39.8%)	
**AUDIT-C score**	5 (4, 7)	5 (4, 7)	5 (3, 7)	**0.011**
Missing	73 (2.2%)	58 (2.2%)	15 (2.2%)	**0.019**
High alcohol	1798 (53.6%)	1460 (54.8%)	338 (48.9%)	
**Liver steatosis (CAP value; dB/m)**	203 (172, 238)	191 (166, 214)	278 (261, 304)	**< 0.001**
**Fibrosis (kPA)**	4.5 (3.8, 5.4)	4.5 (3.8, 5.4)	4.6 (3.8, 5.5)	
Missing	194 (5.8%)	169 (5.6%)	25 (7.5%)	0.38
None	3080 (91.9%)	2454 (92.2%)	626 (90.6%)	
Any	79 (2.4%)	61 (2.3%)	18 (2.6%)	

^a^Hepatic steatosis based on CAP score cut-off values: low (< 248 dB/m, < 11% steatosis) vs mild to severe (248–400 dB/m, ≥11% steatosis) [41]

^b^n = 125 were missing free sugar and total energy intake values

^c^Groups with less than five participants are expressed as *n* < 5 in line with the Avon Longitudinal Study of Parents and Children (ALSPAC) confidentiality policy

^d^Wilcoxon rank sum (equal variance) and Kolmogrov-Smirnov (unequal variance) tests were used for continuous variables. Chi-squared tests were used for categorical variables. Statistically significant *p*-values are bolded

*Abbreviations*: *IQR* Interquartile Range, *CSE* certificate of secondary education, *BMI* Body mass index, *AUDIT-C* Alcohol use disorders identification test consumption, *CAP* controlled attenuation parameter

There was no heterogeneity by sex for any exposure-outcome associations. The results from logistic regression models are presented in Table 2. In both unadjusted and confounder-adjusted analysis, maternal pre-pregnancy overweight (aOR: 1.84, 95% CI: 1.43–2.38), obesity (aOR: 2.73, 95% CI: 1.84–4.03) and more than recommended GWG **(**aOR: 1.30, 95% CI: 1.04–1.64) were positively and independently associated with offspring hepatic steatosis. Being in the highest tertile of percent free sugar consumption was not associated with offspring hepatic steatosis as compared to offspring of mothers in the lowest tertile (aOR: 1.04, 95% CI: 0.82–1.33).

**Table 2:** Associations between maternal factors and offspring hepatic steatosis^a^ at 24 years in the ALSPAC cohort

	1) unadjusted			2) + confounders^d^			3a) + birthweight			3b) + breastfeeding			3c) + 24-year BMI		
	OR	95% CL		OR	95% CL		OR	95% CL		OR	95% CL		OR	95% CL	
Diabetes^b^															
No	Ref			Ref			Ref			Ref			Ref		
Yes	1.49	1.00	2.22	1.39	0.87	2.21	1.36	0.85	2.19	1.34	0.83	2.16	1.12	0.65	1.92
Pre-pregnancy BMI															
Underweight	0.73	0.44	1.22	0.67	0.37	1.20	0.68	0.38	1.23	0.67	0.37	1.23	1.12	0.59	2.11
Normal	Ref			Ref			Ref			Ref			Ref		
Overweight	1.90	1.51	2.40	1.84	1.43	2.38	1.85	1.43	2.40	1.82	1.40	2.36	1.23	0.91	1.65
Obese	3.05	2.14	4.34	2.73	1.84	4.03	2.71	1.82	4.03	2.55	1.69	3.85	0.95	0.59	1.51
Gestational weight gain															
< Rec.	1.15	0.84	1.57	1.11	0.79	1.55	1.11	0.79	1.56	1.06	0.75	1.52	1.25	0.86	1.84
Rec.	Ref			Ref			Ref			Ref			Ref		
> Rec.	1.46	1.18	1.79	1.30	1.04	1.64	1.33	1.06	1.68	1.35	1.07	1.71	1.15	0.89	1.48
Free sugar^c^ tertiles															
1.3–10.4%	Ref			Ref											
10.4–14.3%	1.01	0.82	1.25	1.12	0.88	1.42									
14.3–42.2%	1.02	0.83	1.26	1.04	0.82	1.33									

^a^Hepatic steatosis based on CAP score cut-off values: low (< 248 dB/m, < 11% steatosis) vs mild to severe (248–400 dB/m, ≥11% steatosis) [41]. Sample sizes for each model were 1.Diabetes = 3267; 1.Pre-pregnancy BMI = 3098; 1.GWG = 3032, 1.Free sugar = 3204; 2.Diabetes, BMI, and GWG =2668; 2.Free sugar = 2646; 3a = 2639; 3b = 2522; 3c = 2645

^b^Diabetes is defined as maternal existing diabetes, gestational diabetes, or glycosuria during pregnancy

^c^Free sugars are presented as percent of total energy intake

^d^Confounders include maternal age, highest level of maternal education, maternal smoking in pregnancy, alcohol intake in pregnancy, physical activity in pregnancy, and sex. The maternal exposures (pre-pregnancy BMI, maternal diabetes, and gestational weight gain) were also included as covariates in model 2. The model focused on free sugar exposure, additionally adjusted for total energy intake and did not adjusted for maternal diabetes since those individuals were excluded

*Abbreviations*: *OR* odds ratio, *CL* confidence limits, *BMI* body mass index, *Rec* recommended, *GWG* gestational weight gain

Birthweight (Table 2*, column 3a*) and breastfeeding (Table 2*, column 3b*) were not important mediators in the relationship between maternal factors and offspring steatosis. However, these associations were largely attenuated after adjusting for offspring BMI category at 24 years (Table 2*, column 3c*), indicating that they are largely mediated by offspring adiposity.

In sensitivity analysis, adjusting for hazardous alcohol intake among offspring did not significantly change the associations (Additional file 1, Table S1). The associations between maternal factors and hepatic steatosis were strengthened when we redefined the outcome to severe steatosis, although these associations were also completely mediated by offspring BMI (Additional file 1, Table S2).

## Discussion

Maternal pre-pregnancy overweight, obesity, and more than recommended gestational weight gain were positively and independently associated with offspring hepatic steatosis at 24 years in confounder-adjusted models. These associations were fully mediated by offspring BMI at 24 years.

This is the first study to look at whether the associations between maternal nutritional exposures and offspring hepatic steatosis extend into adulthood and also the first to directly explore associations with maternal free sugar intake. Several previous studies have looked at the associations of maternal BMI, diabetes, and gestational weight gain and offspring NAFLD in children and adolescents [22–24, 49, 50]. (Additional file 1, Table S3). In the RAINE cohort, which included 1170 adolescents of European descent in Australia, NAFLD was associated with maternal obesity and gestational weight gain but not with maternal diabetes, and associations were stronger in females [23]. Breastfeeding for over 6 months, especially when combined with delayed introduction of infant formula milk, had a protective association with NAFLD in this cohort [22]. Breastfeeding, which may influence NAFLD through the gut microbiome, has also been associated with reduced risk of NASH in children and adolescent [51, 52]. In our study, outcomes were not mediated by breastfeeding duration and we did not see differences by sex, except that males had greater prevalence of hepatic steatosis at age 24 years. It is possible we did not see a similar protective effect of breastfeeding on NAFLD because we were not able to further adjust for exclusive breastfeeding.

In the EPOCH cohort based in the US, maternal pre-pregnancy obesity was strongly associated with adolescent hepatic steatosis quantified by MRI and the association was largely mediated by offspring adiposity at time of outcome [50]. Maternal diabetes was not significantly associated with later NAFLD. In contrast to the other cohorts and our study, in the same ALSPAC cohort, Patel et al., found a strong association between maternal diabetes and offspring hepatic steatosis at 17 years that was not mediated by offspring adiposity or BMI [24]. This difference is likely not due to differences in methodology. In both our study and the Patel study, maternal diabetes was defined consistently. While hepatic steatosis was measured differently, by ultrasound at 17 years and controlled attenuation parameter at 24 years, these methods have similar sensitivity and specificity in detecting hepatic steatosis so we would not expect such a large increase in prevalence of hepatic steatosis due to assessment method alone [53]. Our statistical models were also similar. However, at 17 years a smaller subset of the cohort (*n* = 1215 of 4253 that attended the 17-year clinic) was screened for hepatic steatosis, so selection bias is possible. Another possibility for this difference in outcomes may be related to the large increase in prevalence of hepatic steatosis as the cohort reached early adulthood. At 17 years, only 2.1% of a sub-sample of participants had hepatic steatosis, and by 24 years, 20% had hepatic steatosis. It is possible that early-onset steatosis has a different etiology/pathophysiology and the associations are attenuated when overall 24-year prevalence of hepatic steatosis is considered. Adulthood hepatic steatosis may be more associated with adiposity. For example, if the development of NAFLD requires obesity (particularly as individuals age), then the attenuation of estimates by adjusting for BMI is what we would expect to find, and the true association is what we derive from models not adjusting for BMI or adiposity. Future studies should explore the changes in hepatic steatosis that occur from birth to adulthood, and specifically in the ALSPAC cohort from adolescence to adulthood.

Several studies have found associations between maternal obesity/GDM and fetal and infant hepatic steatosis (Additional File 1, Table S3) [6–8, 54, 55]. Modi et al., found intrahepatocellular lipid (IHCL) content in infants increased with maternal BMI [7]. They did not look at the effect of gestational diabetes due to the small number of affected women in the study sample. Brumbaugh et al., found that infants born to obese mothers with GDM had increased IHCL compared with infants born to normal weight mothers [8]. In both studies, IHCL correlated with maternal pre-pregnancy BMI but not with infant subcutaneous adiposity. In the Feeding Study cohort from Italy, mothers of 1 year old children with bright livers, a sign of hepatic steatosis, had greater gestational weight gain compared to mothers of those without bright livers [55]. Logan, et al., found no association of maternal GDM, with infant IHCL [54]. In that study, the GDM mothers had good glycemic control (55% received metformin and/or insulin treatment resulting in a mean (SD) 5.3% (0.3) HbA1c) and little obesity (median BMI = 24.2, IQR (21.7, 30.3), whereas glycemic status was not known in the other studies. Finally, in a retrospective autopsy study, stillborn fetuses of diabetic mothers had 78.8% hepatic steatosis compared to 16.6% in non-diabetic mothers (*p* < 0.0001) regardless of maternal BMI. Further studies of mothers with more prevalent and different types of diabetes are needed to determine whether the association of maternal diabetes with offspring NAFLD is independent of maternal obesity. Additionally, hepatic fat measures in longitudinal birth cohorts ranging from infancy and throughout childhood could clarify the question of whether elevated hepatic fat seen in infancy remains throughout childhood and their relation to overall adiposity.

We found no association between high maternal percent free sugar intake in the third trimester and offspring NAFLD. Women in the lowest tertile had intakes less than 10.4% of total energy and those in the highest tertile had intakes ranging from 14.3 to 42%. The WHO recommended cut-off for intake of free sugars is 10% of total daily energy intake [39]. While some studies have found associations between gestational diet and overall offspring adiposity, none have looked specifically at free sugars in relation to a NAFLD outcome. Animal studies have found strong associations between maternal high fat and sugar diets and offspring hepatic steatosis [14, 15, 56]. The complicated nature of human diet makes it difficult to delineate interactions among nutrients. Another complicating factor is that the most sensitive period of fetal development for hepatic steatosis may be prior to the third trimester. The development of the fetal liver begins at four weeks gestation and is susceptible to fundamental changes to its metabolic pathways through epigenetic changes and mitochondrial dysfunction caused by inflammation due to excess nutrients. Maternal diabetes, obesity, and high fat/sugar diets are all characterized by increased delivery of fuels such as free fatty acids to the developing fetus [57]. Specifically, the liver may be utilized as a site for excess lipid storage, especially prior to 28 weeks gestation when subcutaneous fat storage exponentially increases [58]. Future studies should be designed to accurately measure maternal diet and biomarkers throughout pregnancy to elucidate the exact mechanism through which maternal nutrition can prime offspring for later metabolic dysfunction.

### Strengths and limitations

This study contains the largest sample size to date of maternal factors and offspring NAFLD and is from a population-based longitudinal cohort study followed from pregnancy to young adulthood. Many potential confounding variables were available in the dataset. We also used a validated and accurate tool for hepatic steatosis measurement in adults (CAP score based on transient elastography) [41]. We also were able to account for alcohol intake among participants. It has previously been reported that no participants had medical conditions or were taking medications that could influence hepatic function leading to greater confidence that our measure of hepatic steatosis is primarily capturing those with NAFLD [24]. While hepatic fibrosis was also measured in this study, we were underpowered to look at this as an outcome due to the small number of individuals with fibrosis.

The results of this study may not be generalizable to other populations due to the homogenous nature of the cohort population. Additionally, there was differential loss to follow-up within the cohort which could lead to selection bias. Females and participants with mothers with higher education were more likely to participate in follow-up visits [59]. Many characteristics, including maternal diet and maternal pre-pregnancy weight, were self-reported and are subject to recall bias and social desirability bias. However, it has been shown in this cohort that maternal self-reported weight had a high correlation (*r* = 0.95) with the weight measurement obtained at the first antenatal visit [24]. Maternal diet was collected by an FFQ that was not validated nor designed specifically to measure sugar intake. Measurement of maternal diet throughout pregnancy and with a validated FFQ may have led to different results. Because of the small sample sizes, we were not able to distinguish between types of maternal diabetes. Finally, residual confounding, particularly by lifestyle, is possible.

## Conclusions

Maternal nutritional factors were positively and independently associated with offspring hepatic steatosis at 24 years, however these associations were completely mediated by offspring BMI at 24 years. Importantly, these are all modifiable risk factors and recommendations can be tailored to high-risk women to improve outcomes. Due to the high prevalence of metabolic conditions in pregnant women and the potential for transgenerational amplification of these diseases, further prospective study is needed to better understand the developmental origins of NAFLD and other metabolic conditions [13, 60].

## Supplementary Information

**Additional file 1: Table S1**. Adjusted associations between maternal factors and 24-year hepatic steatosis, also adjusting for hazardous alcohol intake. **Table S2**. Associations between maternal factors and offspring severe hepatic steatosis at 24 years in the ALSPAC cohort. **Table S3**. Summary of literature associating maternal nutrition with offspring hepatic steatosis.

## Data Availability

The data that support the findings of this study are available from the Avon Longitudinal Study of Parents and Children but restrictions apply to the availability of these data, which were used under license for the current study, and so are not publicly available. Data are however available from the authors upon reasonable request and with permission of ALSPAC. Researchers can apply to ALSPAC for use of the data. The study website (http://www.bristol.ac.uk/alspac/researchers/our-data/) contains details of all the data that are available through a fully searchable data dictionary and variable search tool.

## Abbreviations

ALSPAC: Avon Longitudinal Study of Parents and Children
NAFLD: Non-alcoholic fatty liver disease
NASH: Non-alcoholic steatohepatitis
CAP: Controlled attenuation parameter
BMI: Body mass index
GWG: Gestational weight gain
AUDIT-C: Alcohol Use Disorder Identification Test for Consumption
IQR: Interquartile range
CL: Confidence limits
