# Supplementary material for: Associations of maternal diet and nutritional status with offspring hepatic steatosis in the Avon longitudinal study of parents and children
Source: BMC Nutr. 2021 Jul 8;7:28. doi: 10.1186/s40795-021-00433-3 (PMC8265091; doi:10.1186/s40795-021-00433-3)
Supplement: Supplementary file 1 — Additional file 1: Table S1. Adjusted associations between maternal factors and 24-year hepatic steatosis, also adjusting for hazardous alcohol intake. Table S2. Associations between maternal factors and offspring severe hepatic steatosis at 24 years in the ALSPAC cohort. Table S3. Summary of literature associating maternal nutrition with offspring hepatic steatosis. [file 40795_2021_433_MOESM1_ESM.docx]

**Supplemental Tables**

**Table S1. Adjusted^1^ associations between maternal factors and 24-year hepatic steatosis^2^, also adjusting for hazardous alcohol intake.**

|  | **OR** | **95% CL** | |
| --- | --- | --- | --- |
| Diabetes^3^ |  |  |  |
| No | Ref |  |  |
| Yes | 1.27 | 0.79 | 2.05 |
| Pre-pregnancy BMI |  |  |  |
| Underweight | 0.69 | 0.38 | 1.24 |
| Normal | Ref |  |  |
| Overweight | 1.87 | 1.44 | 2.42 |
| Obese | 2.78 | 1.88 | 4.12 |
| Gestational weight gain | |  |  |
| < Recommended | 1.15 | 0.82 | 1.62 |
| Recommended | Ref |  |  |
| > Recommended | 1.31 | 1.04 | 1.65 |

^1^Adjusted for maternal age, highest level of maternal education, maternal smoking in pregnancy, alcohol intake in pregnancy, physical activity in pregnancy, and sex. The maternal exposures (pre-pregnancy BMI, maternal diabetes, and gestational weight gain) were also included as covariates.

^2^Hepatic steatosis based on CAP score cut-off values: low (<248 dB/m, <11% steatosis) vs mild to severe (248-400 dB/m, ≥11% steatosis).^1^

^3^Diabetes is defined as existing, gestational, and glycosuria.

Abbreviations: BMI = body mass index, OR=odds ratio, CL=confidence limits, Ref=reference group.

**Table S2.** **Associations between maternal factors and offspring *severe^1^* hepatic steatosis at 24 years in the ALSPAC cohort**

|  | **1) unadjusted** | | | **2) + confounders^3^** | | | **3a) + birthweight** | | | **3b) + breastfeeding** | | | **3c) + 24-year BMI** | | | **+ Audit-C** | | |
| --- | --- | --- | --- | --- | --- | --- | --- | --- | --- | --- | --- | --- | --- | --- | --- | --- | --- | --- |
|  | **OR** | **95% CL** | | **OR** | **95% CL** | | **OR** | **95% CL** | | **OR** | **95% CL** | | **OR** | **95% CL** | | **OR** | **95% CL** | |
| Diabetes^2^ |  |  |  |  |  |  |  |  |  |  |  |  |  |  |  |  |  |  |
| No | Ref |  |  | Ref |  |  | Ref |  |  | Ref |  |  | Ref |  |  | Ref |  |  |
| Yes | 1.74 | 1.06 | 2.84 | 1.32 | 0.72 | 2.41 | 1.39 | 0.76 | 2.56 | 1.13 | 0.59 | 2.16 | 1.02 | 0.51 | 2.01 | 1.33 | 0.73 | 2.45 |
| Pre-pregnancy BMI |  |  |  |  |  |  |  |  |  |  |  |  |  |  |  |  |  |  |
| Underweight | 0.73 | 0.35 | 1.51 | 0.68 | 0.29 | 1.58 | 0.69 | 0.29 | 1.61 | 0.72 | 0.31 | 1.68 | 1.27 | 0.50 | 3.24 | 0.71 | 0.30 | 1.65 |
| Normal | Ref |  |  | Ref |  |  | Ref |  |  | Ref |  |  | Ref |  |  | Ref |  |  |
| Overweight | 1.84 | 1.35 | 2.50 | 1.78 | 1.27 | 2.49 | 1.83 | 1.31 | 2.57 | 1.69 | 1.20 | 2.39 | 1.11 | 0.76 | 1.63 | 1.80 | 1.28 | 2.53 |
| Obese | 3.89 | 2.60 | 5.81 | 3.63 | 2.32 | 5.69 | 3.74 | 2.36 | 5.92 | 3.36 | 2.09 | 5.41 | 1.16 | 0.68 | 1.98 | 3.68 | 2.35 | 5.78 |
| Gestational weight gain | |  |  |  |  |  |  |  |  |  |  |  |  |  |  |  |  |  |
| < Recommended | 1.05 | 0.67 | 1.63 | 1.11 | 0.69 | 1.80 | 1.09 | 0.67 | 1.76 | 1.20 | 0.74 | 1.96 | 1.25 | 0.73 | 2.14 | 1.17 | 0.72 | 1.90 |
| Recommended | Ref |  |  | Ref |  |  | Ref |  |  | Ref |  |  | Ref |  |  | Ref |  |  |
| > Recommended | 1.59 | 1.20 | 2.11 | 1.42 | 1.03 | 1.96 | 1.46 | 1.06 | 2.02 | 1.48 | 1.07 | 2.06 | 1.20 | 0.84 | 1.71 | 1.46 | 1.06 | 2.02 |
| Free sugar^3^ tertiles |  |  |  |  |  |  |  |  |  |  |  |  |  |  |  |  |  |  |
| 1.3% to 10.4% | Ref |  |  | Ref |  |  |  |  |  |  |  |  |  |  |  |  |  |  |
| 10.4% to 14.3% | 0.97 | 0.73 | 1.28 | 1.04 | 0.76 | 1.42 |  |  |  |  |  |  |  |  |  |  |  |  |
| 14.3% to 42.2% | 0.91 | 0.68 | 1.20 | 0.87 | 0.62 | 1.21 |  |  |  |  |  |  |  |  |  |  |  |  |

^1^Severe steatosis=279-400 dB/m, ≥66% steatosis. Sample sizes for each model were 1.Diabetes = 3,267; 1.Pre-pregnancy BMI=3,098; 1.GWG=3,032, 1.Free sugar=3,204; 2.Diabetes, BMI, and GWG =2668; 2.Free sugar=2,646; 3a = 2,639; 3b=2,522; 3c=2645.

^2^Diabetes is defined as existing, gestational, and glycosuria.

^3^Free sugars are presented in as percent of total energy intake.

^4^Confounders include maternal age, highest level of maternal education, maternal smoking in pregnancy, alcohol intake in pregnancy, physical activity in pregnancy, and sex. The maternal exposures (pre-pregnancy BMI, maternal diabetes, and gestational weight gain) were also included as covariates in model 2. The model focused on free sugar exposure, additionally adjusted for total energy intake and did not adjusted for maternal diabetes since those individuals were excluded.

Abbreviations: BMI = body mass index, UW=underweight, N=Normal, OW=overweight, OB=obese, OR=odds ratio, CL=confidence limits, Ref=reference group, Rec=Recommended.

**Table S3. Summary of literature associating maternal nutrition with offspring hepatic steatosis**

| **Citation** | **Population** | **n** | **Age** | **Method** | **Prevalence^1^** | **Maternal Exposure** | **Association (95% CI)** |
| --- | --- | --- | --- | --- | --- | --- | --- |
| Patel, 2014 | US Hospital | 81 | Fetal | Autopsy | na |  |  |
|  |  |  |  |  |  | Diabetes | +62.1% (p<0.0001) |
|  |  |  |  |  |  | BMI | No association |
| Modi, 2011 | UK Hospital | 105 | Infants | MRI | na |  |  |
|  |  |  |  |  |  | Obesity | β 8.6% (1.1, 16.8) |
|  |  |  |  |  |  | Diabetes | No association |
| Brumbaugh, 2013 | US Hospital | 25 | Infants | MRI | na |  |  |
|  |  |  |  |  |  | Obesity/GDM | +68% |
| Logan, 2016 | UK Hospital | 86 | Infants | MRI | na |  |  |
|  |  |  |  |  |  | GDM | +3.5% (-35.4, 65.6) |
| Bedogni, 2019 | Feeding Study, Italy | 389 | 1 year | Ultrasound | 4.0% |  |  |
|  |  |  |  |  |  | GWG | +2kg GWG |
|  |  |  |  |  |  | Fatty acids^2^ | No difference |
| Santos, 2019 | Generation R, Netherlands | 2354 | 10 yrs | MRI | 2.0% LFF^3^ |  |  |
|  |  |  |  |  |  | BMI | +0.15 (0.11, 0.19) SDS |
|  |  |  |  |  |  | GWG | No association |
| Ayonrinde, 2018 | RAINE cohort, Aus European descent, | 1170 | 17 yrs | Ultrasound | 15.2% |  |  |
|  |  |  |  |  |  | Obesity^4^ | OR 3.46 (1.49, 8.50) |
|  |  |  |  |  |  | GWG^4^ | OR 1.10 (1.04, 1.15) |
|  |  |  |  |  |  | GDM | No association |
| Bellatorre, 2018 | EPOCH cohort  Mixed race/eth, US | 254 | 16 yrs | MRI | 5.9% |  |  |
|  |  |  |  |  |  | Obesity | β 1.59 (0.66, 2.52) |
|  |  |  |  |  |  | Overweight | β 0.39 (-0.44, 1.23) |
|  |  |  |  |  |  | GDM | β -0.46 (-1.37, 0.45) |
| Patel, 2016 | ALSPAC  White, UK | 1215 | 17 yrs | Ultrasound | 2.1% |  |  |
|  |  |  |  |  |  | Obesity | OR 2.72 (1.20, 6.15) |
|  |  |  |  |  |  | Diabetes^5^ | OR 6.74 (2.47, 18.40) |
| Sekkarie | ALSPAC  White, UK | 3354 | 24 yrs | CAP | 20.0% |  |  |
|  |  |  |  |  |  | Overweight | OR 1.84 (1.42, 2.39) |
|  |  |  |  |  |  | Obesity | OR 2.76 (1.85, 4.12) |
|  |  |  |  |  |  | GWG | OR 1.27 (1.01, 1.61) |
|  |  |  |  |  |  | Diabetes^4^ | OR 1.40 (0.88, 2.40) |
|  |  |  |  |  |  | % free sugar | OR 1.02 (0.80, 1.29) |

^1^Prevalence of hepatic steatosis. In infants, intrahepatocellular lipid content (IHCL) content was measured.

^2^Short chain, monounsaturated, polyunsaturated, omega-3, and omega 6 as absolute and % of total fatty acids.

^3^ Median liver fat fraction percent was 2.0% (95% range: 1.2-5.2) in the overall group. Did not present prevalence of hepatic steatosis.

^4^Only in females; GWG was ≥ 6 kilograms in the first trimester. Breastfeeding was independently associated with hepatic steatosis.

^5^Includes pre-existing diabetes, gestational diabetes mellitus, or glycosuria during pregnancy.

Abbreviations: UK=United Kingdom, US=United States, Aus=Australia, yrs=years, MRI=magnetic resonance imaging, CAP=controlled attenuation parameter, GDM=gestational diabetes mellitus, GWG=gestational weight gain, OR=odds ratio, SDS =standard deviation score, LFF = median liver fat fraction, na=not applicable.
